# Comparative genome sequence analysis underscores mycoparasitism as the ancestral life style of *Trichoderma*

**DOI:** 10.1186/gb-2011-12-4-r40

**Published:** 2011-04-18

**Authors:** Christian P Kubicek, Alfredo Herrera-Estrella, Verena Seidl-Seiboth, Diego A Martinez, Irina S Druzhinina, Michael Thon, Susanne Zeilinger, Sergio Casas-Flores, Benjamin A Horwitz, Prasun K Mukherjee, Mala Mukherjee, László Kredics, Luis D Alcaraz, Andrea Aerts, Zsuzsanna Antal, Lea Atanasova, Mayte G Cervantes-Badillo, Jean Challacombe, Olga Chertkov, Kevin McCluskey, Fanny Coulpier, Nandan Deshpande, Hans von Döhren, Daniel J Ebbole, Edgardo U Esquivel-Naranjo, Erzsébet Fekete, Michel Flipphi, Fabian Glaser, Elida Y Gómez-Rodríguez, Sabine Gruber, Cliff Han, Bernard Henrissat, Rosa Hermosa, Miguel Hernández-Oñate, Levente Karaffa, Idit Kosti, Stéphane Le Crom, Erika Lindquist, Susan Lucas, Mette Lübeck, Peter S Lübeck, Antoine Margeot, Benjamin Metz, Monica Misra, Helena Nevalainen, Markus Omann, Nicolle Packer, Giancarlo Perrone, Edith E Uresti-Rivera, Asaf Salamov, Monika Schmoll, Bernhard Seiboth, Harris Shapiro, Serenella Sukno, Juan Antonio Tamayo-Ramos, Doris Tisch, Aric Wiest, Heather H Wilkinson, Michael Zhang, Pedro M Coutinho, Charles M Kenerley, Enrique Monte, Scott E Baker, Igor V Grigoriev

**Affiliations:** 1 Area Gene Technology and Applied Biochemistry, Institute of Chemical Engineering Vienna University of Technology, Getreidemarkt 9, 1060 Vienna, Austria; 2 Laboratorio Nacional de Genómica para la Biodiversidad, Cinvestav Campus Guanajuato, Km. 9.6 Libramiento Norte, Carretera Irapuato-León, 36821 Irapuato, Mexico; 3 Broad Institute of MIT and Harvard, 301 Binney St, Cambridge, MA 02142, USA; 4 Centro Hispanoluso de Investigaciones Agrarias (CIALE), Department of Microbiology and Genetics, University of Salamanca, Calle Del Duero, 12, Villamayor 37185, Spain; 5 División de Biología Molecular, Instituto Potosino de Investigación Científica y Tecnológica, Camino a la Presa San José, No. 2055, Colonia Lomas 4a Sección, San Luis Potosí, SLP., 78216, México; 6 Department of Biology, Technion - Israel Institute of Technology, Neve Shaanan Campus, Technion City, Haifa, 32000, Israel; 7 Nuclear Agriculture and Biotechnology Division, Bhabha Atomic Research Centre, Trombay, Mumbai 400085, India; 8 Department of Microbiology, Faculty of Science and Informatics, University of Szeged, Közép fasor 52, Szeged, H-6726, Hungary; 9 DOE Joint Genome Institute, 2800 Mitchell Drive, Walnut Creek, CA 94598, USA; 10 School of Biological Sciences, University of Missouri- Kansas City, 5007 Rockhill Road, Kansas City, MO 64110, USA; 11 Institut de Biologie de l'École normale supérieure (IBENS), Institut National de la Santé et de la Recherche Médicale U1024, Centre National de la Recherche Scientifique UMR8197, 46, rue d'Ulm, Paris 75005, France; 12 Chemistry and Biomolecular Sciences, Macquarie University, Research Park Drive Building F7B, North Ryde, Sydney, NSW 2109, Australia; 13 TU Berlin, Institut für Chemie, FG Biochemie und Molekulare Biologie OE2, Franklinstr. 29, 10587 Berlin, Germany; 14 Department of Plant Pathology and Microbiology Building 0444, Nagle Street, Texas A&M University College Station, TX 77843, USA; 15 Department of Biochemical Engineering, Faculty of Science and Technology, University of Debrecen, Egyetem tér 1, Debrecen, H-4010, Hungary; 16 Instituto de Agroquímica y Tecnología de Alimentos, Consejo Superior de Investigaciones Científicas, Apartado de Correos 73, Burjassot (Valencia) E-46100, Spain; 17 Architecture et Fonction des Macromolécules Biologiques, UMR6098, CNRS, Université de la Méditerranée, Case 932, 163 Avenue de Luminy, 13288 Marseille 13288, France; 18 Department of Biotechnology, Chemistry and Environmental Engineering, Aalborg University, Lautrupvang 15, DK-2750 Ballerup, Denmark; 19 Biotechnology Department, IFP Energies nouvelles, 1-4 avenue de Bois Préau, Rueil-Malmaison, 92852, France; 20 Institute of Sciences of Food Production (ISPA), National Research Council (CNR), Via Amendola 122/O, 70126 Bari, Italy; 21 Wageningen University, Systems and Synthetic Biology, Fungal Systems Biology Group, Dreijenplein 10, 6703 HB Wageningen, The Netherlands; 22 Chemical and Biological Process Development Group, Pacific Northwest National Laboratory, 902 Battelle Boulevard, Richland, WA 99352, USA

## Abstract

**Background:**

Mycoparasitism, a lifestyle where one fungus is parasitic on another fungus, has special relevance when the prey is a plant pathogen, providing a strategy for biological control of pests for plant protection. Probably, the most studied biocontrol agents are species of the genus *Hypocrea*/*Trichoderma*.

**Results:**

Here we report an analysis of the genome sequences of the two biocontrol species *Trichoderma atroviride *(teleomorph *Hypocrea atroviridis*) and *Trichoderma virens *(formerly *Gliocladium virens*, teleomorph *Hypocrea virens*), and a comparison with *Trichoderma reesei *(teleomorph *Hypocrea jecorina*). These three *Trichoderma *species display a remarkable conservation of gene order (78 to 96%), and a lack of active mobile elements probably due to repeat-induced point mutation. Several gene families are expanded in the two mycoparasitic species relative to *T. reesei *or other ascomycetes, and are overrepresented in non-syntenic genome regions. A phylogenetic analysis shows that *T. reesei *and *T. virens *are derived relative to *T. atroviride*. The mycoparasitism-specific genes thus arose in a common *Trichoderma *ancestor but were subsequently lost in *T. reesei*.

**Conclusions:**

The data offer a better understanding of mycoparasitism, and thus enforce the development of improved biocontrol strains for efficient and environmentally friendly protection of plants.

## Background

Mycoparasitism is the phenomenon whereby one fungus is parasitic on another fungus, a lifestyle that can be dated to at least 400 million years ago by fossil evidence [1]. This has special relevance when the prey is a plant pathogen, providing a strategy for biological control of pests for plant protection ('biocontrol'). The movement toward environmentally friendly agricultural practices over the past two decades has thus accelerated research in the use of biocontrol fungi [2]. Probably the most studied biocontrol agents are species of the genus *Hypocrea*/*Trichoderma, Trichoderma atroviride *(*Ta*) and *Trichoderma virens *(*Tv*) - teleomorphs *Hypocrea atroviridis *and *Hypocrea virens*, respectively - being among the best mycoparasitic biocontrol agents used in agriculture [3]. The beneficial effects of *Trichoderma *spp. on plants comprise traits such as the ability to antagonize soil-borne pathogens by a combination of enzymatic lysis, secretion of antibiotics, and competition for space and substrates [4,5]. In addition, it is now known that some *Trichoderma *biocontrol strains also interact intimately with plant roots, colonizing the outer epidermis layers, and acting as opportunistic, avirulent plant symbionts [6].

Science-based improvement of biocontrol agents for agricultural applications requires an understanding of the biological principles of their actions. So far, some of the molecular aspects - such as the regulation and role of cell wall hydrolytic enzymes and antagonistic secondary metabolites - have been studied in *Trichoderma *[3-5]. More comprehensive analyses (for example, by the use of subtractive hybridization techniques, proteomics or EST approaches) have also been performed with different *Trichoderma *species, but the interpretation of the data obtained is complicated by the lack of genome sequence information for the species used (reviewed in [7]).

Recently, the genome of another *Trichoderma, Trichoderma reesei *(*Tr*, teleomorph *H. jecorina*), which has a saprotrophic lifestyle and is an industrial producer of plant biomass hydrolyzing enzymes, has been sequenced and analyzed [8]. Here we report the genome sequencing and comparative analysis of two widely used biocontrol species of *Trichoderma*, that is, *Ta *and *Tv*. These two were chosen because they are distantly related to *Tr *[9] and represent well defined phylogenetic species [10,11], in contrast to *Trichoderma harzianum sensu lato*, which is also commonly used in biocontrol but constitutes a complex of several cryptic species [12].

## Results

### Properties of the *T. atroviride *and *T. virens *genomes

**Figure 1:**
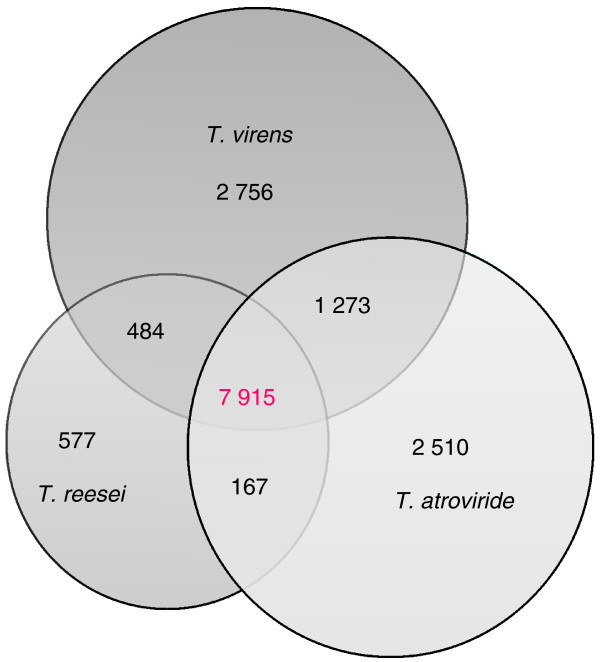
**Distribution of orthologues of *T. atroviride, T. virens *and *T. reesei***. The Venn diagram shows the distribution found for the three species of *Trichoderma*.

The genomes of *Ta *IMI 206040 and *Tv *Gv29-8 were sequenced using a whole genome shotgun approach to approximately eight-fold coverage and further improved using finishing reactions and gap closing. Their genome sizes were 36.1 (*Ta*) and 38.8 Mbp (*Tv*), and thus larger than the 34 Mbp determined for the genome of *Tr *[8]. Gene modeling, using a combination of homology and *ab initio *methods, yielded 11,865 gene models for *Ta *and 12,428 gene models for *Tv*, respectively (Table 1), both greater than the estimate for *Tr *(9,143). As shown in Figure 1, the vast majority of the genes (7,915) occur in all three *Trichoderma *species. Yet *Tv *and *Ta *contain about 2,756 and 2,510 genes, respectively, that have no true orthologue in any of the other species, whereas *Tr *has only 577 unique genes. *Tv *and *Ta *share 1,273 orthologues that are not present in *Tr*, which could thus be part of the factors that make *Ta *and *Tv *mycoparasites (for analysis, see below).

**Table 1:** Genome assembly and annotation statistics

	*T. atroviride*	*T. virens*	*T. reesei*
Genome size, Mbp	36.1	38.8	34.1
Coverage	8.26×	8.05×	9.00×
Assembly gaps, Mbp	0.1 (0.16%)	0.2(0.4%)	0.05 (0.1%)
Number of scaffolds	50	135	89
Number of predicted genes	11865	12518	9143
Gene length, bp	1747.06	1710.05	1793,25
Protein length, amino acids	471.54	478.69	492,27
Exons per gene	2,93	2,98	3,06
Exon length, bp	528.17	506.13	507,81
Intron length, bp	104.20	104.95	119,64
Supported by homology, NR	10,219 (92%)	10,915 (94%)	8409 (92%)
Supported by homology, Swissprot	8,367(75%)	8,773 (75%)	6763 (74%)
Has PFAM domain	5,883 (53%)	6,267 (54%)	5096 (56%)

NR, non-redundant database; PFAM, protein families.

With respect to other ascomycetes, *Tr, Ta and Tv *share 6,306/7,091, 6,515/7,549, and 6,564/7,733 orthologues with *N. crassa *and *Gibberella zeae*, respectively. Thus, approximately a third of the genes in the three *Trichoderma *species are not shared in even the relatively close relative *G. zeae *and are thus unique to *Trichoderma*.

### Genome synteny

A comparison of the genomic organization of genes in *Ta, Tv *and *Tr *showed that most genes are in synteny: only 367 (4%) genes of *Tr*, but 2,515 (22%) of genes of *Tv *and 2,690 (21%) genes of *Ta *are located in non-syntenic regions (identified as a break in synteny by a series of three or more genes (Table 2); a global visual survey can be obtained at the genome websites of the three *Trichoderma *species (see Materials and methods) by clicking 'Synteny' and 'Dot Plot'). As observed for other fungal genomes [13-15], extensive rearrangements have occurred since the separation of these three fungi but with the prevalence of small inversions [16]. The numbers of the synteny blocks increased with their decreased size, compatible with the random breakage model [14] as in aspergilli [15,17]. Sequence identity between syntenic orthologs was 70% (*Tr *versus *Ta*), 78% (*Tr *versus *Tv*), and 74% (*Tv *versus *Ta*), values that are similar to those calculated for aspergilli (for example, *Aspergillus fumigatus *versus *Aspergillus niger *(69%) and versus *Aspergillus nidulans *(68%) and comparable to those between fish and man [17,18].

**Table 2:** Occurrence of orthologues, paralogues and singletons in the genomes of the three *Trichoderma *spp

Genome	Synteny	Total genes	**Orthologs**^**a**^	Non-orthologs	***P*-value**^**b**^
*T. atroviride*	Syntenic	9,350	7,326	2,024	2.2e-16
	Non-syntenic	2,515	1,265	1,250	
*T. virens*	Syntenic	9,828	7,326	2,502	2.2e-16
	Non-syntenic	2,690	1,532	1,158	
*T. reesei*	Syntenic	8,776	7,326	1,450	2.2e-16
	Non-syntenic	367	153	214	

^a^Orthologs that are in all three genomes. ^b^Null hypothesis that the proportion of non-orthologs that are syntenic is less than the proportion of non-orthologs that are non-syntenic. *P*-value: null hypothesis that the proportion of paralogs that are syntenic is less than the proportion of paralogs that are non-syntenic.

### Transposons

A scan of the genome sequences with the *de novo *repeat finding program 'Piler' [19] - which can detect repetitive elements that are least 400 bp in length, have more than 92% identity and are present in at least three copies - was unsuccessful at detecting repetitive elements. The lack of repetitive elements detected in this analysis is unusual in filamentous fungi and suggests that, like the *Tr *genome [8], but unlike most other filamentous fungi, the *Ta *and *Tv *genomes lack a significant repetitive DNA component.

Because of the paucity of transposable elements (TEs) in the *Trichoderma *genomes, we wondered whether simple sequence repeats and minisatellite sequences may also be rare. To this end, we surveyed the genomes of the *Trichoderma *species using the program Tandem Repeat Finder [20]. We also included the genomes of three additional members of the Sordariomycetes and one of the Eurotiomycetes as reference (Table S1 in Additional file 1). Satellite DNA content varied from as little as 2,371 loci (0.53% of the genome) in *A. nidulans *to 9,893 (1.46% of the genome) in *Neurospora crassa*. Satellite DNA content of the *Trichoderma *genomes ranged from 5,249 (0.94%) in *Ta *to 7,743 (1.54%) in *Tr*. Since these values are within the range that we found in the reference species, we conclude that there is no unusual variation in the satellite DNA content of the *Trichoderma *genomes.

We also scanned the genomes with RepeatMasker and RepeatProteinMask [21] to identify sequences with similarity to known TEs from other organisms. Thereby, sequences with significant similarity to known TEs from other eukaryotes were identified (Table 3). In most cases, the TE families that we detected were fragmented and highly divergent from one another, suggesting that they did not arise from recent transposition events. Based on these results, we conclude that no extant, functional TEs exist in the *Trichoderma *genomes. The presence of ancient, degenerate TE copies suggests that *Trichoderma *species are occasionally subject to infection, or invasion by TEs, but that the TEs are rapidly rendered unable to replicate and rapidly accumulate mutations.

**Table 3:** The major classes of transposable elements found in the *Trichoderma *genomes

	*T. atroviridae*		*T. reesei*		*T. virens*	
	
Class	Copy number	Total length (bp)	Copy number	Total length (bp)	Copy number	Total length (bp)
DNA	372	39,899	446	50,448	370	52,358
LTR	533	64,534	559	76,482	541	67,484
Helitrons	40	9,235	45	9,962	34	8,547
LINE	561	65,202	530	54,928	349	59,414
Total^a^		178,870 (0.49%)		191,820 (0.57%)		187,803 (0.48%)

^a^Total in base pairs and percentage of genome of transposable elements found in the genomes. LINE, long interspersed nuclear element; LTR, long terminal repeat.

### Evidence for the operation of repeat-induced point mutation in *Trichoderma*

The paucity of transposons in *Trichoderma *could be due to repeat-induced point mutation (RIP), a gene silencing mechanism. In* N. crassa* and many other filamentous fungi, RIP preferentially acts on CA dinucleotides, changing them to TA [22]. Thus, in sequences that have been subject to RIP, one should expect to find a decrease in the proportion of CA dinucleotides and its complement dinucleotide TG as well as a corresponding increase in the proportion of TA dinucleotides. The RIP indices TA/AT and (CA + TG)/(AC + GT) developed by Margolin *et al. *[22] can be used to detect sequences that have been subject to RIP. Sequences that have been subjected to RIP are expected to have a high TA/AT ratio and low (CA + TG)/(AC + GT) ratio, with values >0.89 and <1.03, respectively, being indicative of RIP [22,23].

To identify evidence for RIP in the TE sequences, we computed RIP indices for four of the most prevalent TE families in each of the three species (Table 4). Since many of the sequences are very short, we computed the sum of the dinucleotide values within each TE family within each species, and used the sums to compute the RIP ratios. In only one of the 12 families did we find that both RIP indices were within the ranges that are typically used as criteria for RIP. Most of the TE sequences that we identified in the *Trichoderma *genomes are highly degenerate and have likely continued to accumulate mutations after the RIP process has acted on them. We suspect that these mutations have masked the underlying bias in dinucleotide frequencies, making the RIP indices ineffective at identifying the presence of RIP. To overcome this, we also prepared manually curated multiple sequence alignments of the TE families, selecting only sequences that had the highest sequence similarity, and thus should represent the most recent transposon insertion events in the genomes. We were able to prepare curated alignments for all four of the test TE families of *Tr *and *Tv *only for the long terminal repeat element Gypsy and the long interpersed nuclear element R1 in *Ta *(Table S2 in Additional file 1). Among DNA sequences that make up these ten alignments, we detected RIP indices within the parameters that are indicative of RIP in seven alignments. In addition, all seven alignments have high transition/transversion ratios, as is expected in sequences that are subject to RIP.

**Table 4:** Repeat-induced point mutation ratios for four of the most abundant transposable element families in the three *Trichoderma *species

Sequence	TA/AT ratio	CT+AT/AC+GT ratio	**RIP**^**a**^
***T. atroviride***	0.70	1.35	
LTR Copia	0.42	1.50	
LTR Gypsy	0.97	1.21	
LINE R1	1.86	1.67	
LINE Tad1	0.82	1.32	
***T. reesei***	0.71	1.28	
LTR Copia	1.04	1.31	
LTR Gypsy	1.01	1.28	
LINE R1	0.99	2.40	
LINE Tad1	0.33	1.30	
***T. virens***	0.71	1.33	
LTR Copia	0.77	1.48	
LTR Gypsy	0.95	1.16	
LINE R1	0.75	2.14	
LINE Tad1	1.33	0.99	*

^a^The asterisk indicates the family Tad1 from *T. virens *in which the RIP ratios fall within values that are typically associated with RIP. LINE, long interspersed nuclear element; LTR, long terminal repeat; RIP, repeat-induced point mutation; TE, transposable element.

Finally, screening of the genome sequences of *Tr, Ta *and *Tv *identified orthologues of all genes required for RIP in *N. crassa *(Table 5).

**Table 5:** Presence of genes in *Trichoderma *known to be required in *N. crassa *for repeat-induced point mutation

***N. crassa protein***^**a**^	**Accession number**^**a**^	Function^a^	*Trichoderma *orthologue (ID number)		
			*T. atroviride*	*T. virens*	*T. reesei*
**RIP**					
RID	XP_959047.1	Putative DMT, essential for RIP and for MIP			
Dim-5	XP_957479.2	Histone 3-K9 HMT essential for RIP; RdRP	152017	55211	515216
**Quelling**					
QDE-1	XP_959047.1	RdRP, essential for quelling	361	64774	67742
QDE-2	XP_960365.2	Argonaute-like protein, essential for quelling	79413	20883	49832
QDE-3	XP_964030.2	RecQ helicase, essential for quelling	91316	30057	102458
DCL1	XP_961898.1	Dicer-like protein, involved in quelling	20162	20212	69494
DCL2	XP_963538.2	Dicer-like protein, involved in quelling	318	47151	79823
QIP	CAP68960.1	Putative exonuclease protein, involved in quelling	14588	41043	57424
**MSUD**					
SAD-1	XP_964248.2	RdRP essential for MSUD	465	28428	103470
SAD-2	XP_961084.1	Essential for MSUD	No	No	No

^a^*N. crassa *gene information and abbreviations taken from [36]. DMT, cytosine DNA methyltransferase; HMT, histone methyltransferase; MIP, methylation induced premeotically; MSUD, meiotic silencing of unpaired DNA; RdRP, RNA-dependent RNA-polymerase.

### Paralogous gene expansion in *T. atroviride *and *T. virens*

We used Marcov cluster algorithm (MCL) analysis [24] and included ten additional ascomycete genomes present in the Joint Genome Institute (JGI) genome database (including Eurotiomycetes, Sordariomycetes and Dothidiomycetes) to identify paralogous gene families that have become expanded either in all three *Trichoderma *species or only in the two mycoparasitic *Trichoderma *species. Forty-six such families were identified for all three species, of which 26 were expanded only in *Ta *and *Tv*. The largest paralogous expansions in all three *Trichoderma *species have occurred with genes encoding Zn(2)Cys(6) transcription factors, solute transporters of the major facilitator superfamily, short chain alcohol dehydrogenases, S8 peptidases and proteins bearing ankyrin domains (Table 6). The most expanded protein sets, however, were those that were considerably smaller in *Tr *(*P *< 0.05). These included ankyrin proteins with CCHC zinc finger domains, proteins with WD40, heteroincompatibility (HET) and NACHT domains, NAD-dependent epimerases, and sugar transporters.

**Table 6:** Major paralogous gene expansions in Trichoderma

PFAM domain	*T. reesei*	*T. virens*	*T. atroviride*	**Other fungi**^**a**^
**Unknown protein with ankyrin (PF00023), CCHC zinc finger (PF00098; C-X2-C-X4-H-X4-C) and purine nucleoside phosphorylase domain (01048)**	19	***38***	***45***	4
**Zn(II)Cys6 transcription factor (00172) cluster 1-5**	20	***43***	***42***	5,1
Peptidase S8 subtilisin cluster 1-4	10	***33***	***36***	9,6
**Unknown protein with WD40, NACHT and HET domain**	13	***38***	***35***	3,4
**Short chain alcohol dehydrogenase (PF00106) cluster 1 and 2**	20	32	34	4,7
**Unknown protein family 1-4**	12	***25***	***28***	5
**NAD-dependent epimerase (PFAM 01370)**	10	***21***	***23***	5,8
**Isoflavon reductase, plus PAPA-1 (INO80 complex subunit B), epimerase and Nmr1 domain**	9	***18***	***19***	6
**Ankyrin domain protein**	10	17	19	8
**Sugar transporters**	11	24	18	10,8
**GH18 chitinases**	6	***11***	***16***	2
**Protein kinase (00069) plus TPR domain**	2	***24***	***15***	4,7
Unknown major facilitator subfamily (PF07690) domain	9	15	15	5,5
F-box domain protein	7	10	11	1,7
Ankyrin domain protein with protein kinase domain	6	8	11	2,7
Amidase	4	11	11	2,8
Epoxide hydrolase (PF06441) plus AB hydrolase_1 (PF00561)	5	14	11	3,2
FAD_binding_4, plus HET and berberine bridge enzymes (08031) domain	5	13	11	6,1
FMN oxidoreductases	2	8	10	2,5
Unknown protein with DUF84 (NTPase) and NmrA domain	5	19	10	3,7
Protein with GST_N and GST_C domains	6	12	10	4,6
**Class II hydrophobins**	6	8	9	1,1
**Proteins with LysM binding domains**	6	7	9	1,2
**Unknown protein family with NmrA domain**	2	***11***	***8***	0,2
**Pro_CA**	5	9	8	1,3
**WD40 domain protein**	5	11	8	2,2
**C2H2 transcription factors**	1	***5***	***7***	1,4
GFO_IDH_MocA (01408 and 02894) oxidoreductase	3	9	7	1,5
**Protein kinase (00069)**	4	6	6	0,7
**Nonribosomal peptide synthase**	3	4	5	1
**SSCP ceratoplatanin-family**	3	4	5	1
**GH75 chitosanase**	3	5	5	1,1
**SNF2, DEAD box helicase**	3	5	5	1,3
Nitrilase	3	6	5	2,2
**GH65 trehalose or maltose phosphorylase (PFAM 03632)**	4	4	4	0,8
AAA-family ATPase (PF00004)	4	3	4	1
Pyridoxal phosphate dependent decarboxylase (00282)	2	3	4	1,2
Unknown protein	3	4	4	1,3

^a^Results are from MCL analysis of the three *Trichoderma *species (*Tr, Ta, Tv*) and mean values from ten other ascomycetes whose genomes are present in the JGI database [63]. Eurotiomycetes: *Aspergillus carbonarius, Aspergillus niger*. Sordariomycetes: *Thielavia terrerstris, Chaetomium globosum, Cryphonectria parasitica, Neurospora discreta, Neurospora tetrasperma*. Dothidiomycetes: *Mycospherella graminicola, Mycospherella fijiensis, Cochliobolus heterostrophus*. The number of genes present in the "other fungi" is averaged. Data were selected from a total of 28,919 clusters, average cluster number 5.8 (standard deviation 15.73). PFAM categories printed in bold specify those that are significantly (*P *< 0.05) expanded in all three *Trichoderma *species; numbers in bold and italics specify genes that are significantly more abundant in *Ta *and *Tv *versus *Tr *(*P *< 0.05). GH, glycosyl hydrolase family; GST, glutahionine-S transferase; SSCP, small secreted cystein-rich protein.

### Genes with possible relevance for mycoparasitism are expanded in *Trichoderma*

Mycoparasitism depends on a combination of events that include lysis of the prey's cell walls [3,4,7]. The necessity to degrade the carbohydrate armor of the prey's hyphae is reflected in an abundance of chitinolytic enzymes (composing most of the CAZy (Carbohydrate-Active enZYmes database) glycoside hydrolase (GH) family GH18 fungal proteins along with more rare endo-β-N-acetylglucosaminidases) and β-1,3-glucanases (families GH17, GH55, GH64, and GH81) in *Trichoderma *relative to other fungi. Family GH18, containing enzymes involved in chitin degradation, is also strongly expanded in *Trichoderma*, but particularly in *Tv *and *Ta*, which contain the highest number of chitinolytic enzymes of all described fungi (Table 7). Chitin is a substantial component of fungal cell walls and chitinases are therefore an integral part of the mycoparasitic attack [3,25]. It is conspicuous that not only was the number of chitinolytic enzymes elevated but that many of these chitinases contain carbohydrate binding domains (CBMs). Mycoparasitic *Trichoderma *species are particularly rich in subgroup B chitinases that contain CBM1 modules, historically described as cellulose binding modules, but binding to chitin has also been demonstrated [26]. *Tv *and *Ta *each have a total of five CBM1-containing GH18 enzymes. Subgroup C chitinases possess CBM18 (chitin-binding) and CBM50 modules (also known as LysM modules; described as peptidoglycan- and chitin-binding modules). Interestingly, CBM50 modules in *Trichoderma *are found not only in chitinases but also frequently as multiple copies in proteins containing a signal peptide, but with no identifiable hydrolase domain. In most cases these genes can be found adjacent to chitinases in the genome.

**Table 7:** Glycosyl hydrolase families involved in chitin/chitosan and β-1,3 glucan hydrolysis that are expanded in mycoparasitic *Trichoderma *species

		Glycosyl hydrolase family						
			
		**Chitin/chitosan**^**a**^		**ß-glucan**^**a**^				**Total ß-glucan**^**b**^
		
	Taxonomy	GH18	GH75	GH17	GH55	GH64	GH81	217
***Trichoderma atroviride***	S	**29**	**5**	5	**8**	**3**	2	18
***Trichoderma virens***	S	**36**	**5**	4	**10**	**3**	1	18
***Trichoderma reesei***	S	**20**	**3**	4	**6**	**3**	2	15
**Pezizomycota**								
*Nectria haematococca*	S	*28*	2	6	5	2	1	14
*Fusarium graminearum*	S	19	1	6	3	2	1	12
*Neurospora crassa*	S	12	1	4	6	2	1	13
*Podospora anserina*	S	20	1	4	7	1	1	13
*Magnaporthe grisea*	S	14	1	7	3	1	2	13
*Aspergillus nidulans*	E	19	2	5	6	0	1	12
*Aspergillus niger*	E	14	2	5	3	0	1	9
*Penicillium chrysogenum*	E	9	1	5	3	2	1	11
*Tuber melanosporum*	P	5	1	4	2	0	*3*	9
**Other ascomycetes**								
*Saccharomyces cerevisiae*	SM	2	0	4	0	0	2	6
*Schizosaccharomyces pombe*	SS	1	0	1	0	0	1	2
**Basidiomycota**								
*Phanerochaete chrysosporium*	A	11	0	2	2	0	0	4
*Laccaria bicolor*	A	10	0	4	2	0	0	6
*Postia placenta*	A	20	0	4	6	0	0	10

^a^Main substrates for the respective enzyme families. ^b^Number of all enzymes that can act on ß-glucan as a substrate. Taxonomy abbreviations: S, Sordariomycetes; E, Eurotiomycetes; P, Pezizomycetes; S, Saccharomycetes; SS, Schizosaccharomycetes; A, Agaricomycetes. The bold numbers indicate glycosyl hydrolase (GH) families that have a statistically significant expansion in *Trichoderma *(*P *< 0.05) or *Ta *and *Tv *(GH18). This support was obtained only when *N. haematococca *and *T. melanosporum *were not included in the analysis of GH18 and GH81, respectively.

Together with the expanded presence of chitinases, the number of GH75 chitosanases is also significantly expanded in all three analyzed *Trichoderma *species. As with plant pathogenic fungi [27,28], we have also observed an expansion of plant cell wall degrading enzyme gene families. A full account of all the carbohydrate active enzymes is presented in Tables S3 to S8 in Additional file 1. Additional details about the *Trichoderma *CAZome (the genome-wide inventory of CAZy) are given in Chapter 1 of Additional file 2.

Another class of genes of possible relevance to mycoparasitism are those involved in the formation of secondary metabolites (Chapter 2 of Additional file 2). With respect to these, the three *Trichoderma *species contained a varying assortment of non-ribosomal peptide synthetases (NRPS) and polyketide synthases (PKS) (Table 8; see also Tables S9 and S10 in Additional file 1). While *Tr *(10 NRPS, 11 PKS and 2 NRPS/PKS fusion genes [8]) ranked at the lower end when compared to other ascomycetes, *Tv *exhibited the highest number (50) of PKS, NRPS and PKS-NRPS fusion genes, mainly due to the abundance of NRPS genes (28, twice as much as in other fungi). A phylogenetic analysis showed that this was due to recent duplications of genes encoding cyclodipeptide synthases, cyclosporin/enniatin synthase-like proteins, and NRPS-hybrid proteins (Figure S1 in Additional file 3). Most of the secondary metabolite gene clusters present in *Tr *were also found in *Tv *and *Ta*, but about half of the genes remaining in the latter two are unique for the respective species, and are localized on non-syntenic islands of the genome (see below). Within the NRPS, all three *Trichoderma *species contained two peptaibol synthases, one for short (10 to 14 amino acids) and one for long (18 to 25 amino acids) peptaibols. The genes encoding long peptaibol synthetase lack introns and produce an mRNA that is 60 to 80 kb long that encodes proteins of approximately 25,000 amino acids, the largest fungal proteins known.

**Table 8:** The number of polyketide synthases and non-ribosomal peptide synthetases of *Trichoderma *compared to other fungi

Fungal species	PKS	NRPS	PKS-NRPS NRPS-PKS	Total
***Trichoderma virens***	**18**	**28**	**4**	**50**
*Aspergillus oryzae*	26	14	4	44
*Aspergillus nidulans*	26	13	1	40
*Cochliobolus heterostrophus*	23	11	2	36
***Trichoderma atroviride***	**18**	**16**	**1**	**35**
*Magnaporthe oryzae*	20	6	8	34
*Fusarium graminearum*	14	19	1	34
*Gibberella moniliformis*	12	16	3	31
*Botryotinia fuckeliana*	17	10	2	29
*Aspergillus fumigatus*	13	13	1	27
*Nectria haematococca*	12	12	1	25
***Trichoderma reesei***	**11**	**10**	**2**	**23**
*Neurospora crassa*	7	3	0	10

Besides PKS and NRPS, *Ta *and *Tv *have further augmented their antibiotic arsenal with genes for cytolytic peptides such as aegerolysins, pore-forming cytolysins typically present in bacteria, fungi and plants, yeast-like killer toxins and cyanovirins (Chapter 2 of Additional file 2). In addition, we found two high molecular weight toxins in *Ta *and *Tv *that bear high similarity (E-value 0 for 97% coverage) to the Tc ('toxin complex') toxins of *Photorhabdus luminescens*, a bacterium that is mutualistic with entomophagous nematodes [29] (Table S11 in Additional file 1). Apart from *Trichoderma*, they are also present in *G. zeae *and *Podospora anserina*. Yet there may be several more secondary metabolite genes to be detected: *Trichoderma *species contain expanded arrays of cytochrome P450 CYP4/CYP19/CYP26 subfamilies (Table S12 in Additional file 1), and of soluble epoxide hydrolases that could act on the epoxides produced by the latter (Figure S2 in Additional file 3).

The *Hypocrea*/*Trichoderma *genomes also contain an abundant arsenal of putatively secreted proteins of 300 amino acids or less that contain at least four cysteine residues (small secreted cysteine-rich proteins (SSCPs); Chapter 3 of Additional file 2). They contained both unique and shared sets of SSCPs, with a higher complexity in *Tv *and *Ta *than in *Tr *(Table S13 in Additional file 1).

### Genes present in *T. atroviride *and *T. virens *but not in *T. reesei*

As mentioned above, 1,273 orthologous genes were shared between *Ta *and *Tv *but absent from *Tr*. When the encoded proteins were classified according to their PFAM domains, fungal specific Zn(2)Cys(6) transcription factors (PF00172, PF04082) and solute transporters (PF07690, PF00083), all of unknown function, were most abundant (Table S14 in Additional file 1). However, the presence of several PFAM groups of oxidoreductases and monooxygenases, and of enzymes for AMP activation of acids, phosphopathetheine attachment and synthesis of isoquinoline alkaloids was also intriguing. This suggests that *Ta *and *Tv *may contain an as yet undiscovered reservoir of secondary metabolites that may contribute to their success as mycoparasites.

We also annotated the 577 genes that are unique in *T. reesei*: the vast majority of them (465; 80.6%) encoded proteins of unknown function or proteins with no homologues in other fungi. The remaining identified 112 genes exhibited no significant abundance in particular groups, except for four Zn(2)Cys(6) transcription factors, four ankyrins, four HET-domain proteins and three WD40-domain containing proteins.

### Evolution of the non-syntenic regions

A search for overrepresentation of PFAM domains and Gene Ontology terms in the non-syntenic regions described above revealed that all retroposon hot spot repeat domains [30] are found in the non-syntenic regions. In most eukaryotes, these regions are located in subtelomeric areas that exhibit a high recombination frequency [31]. In addition, the genes for the protein families in *Tv *and *Ta *that were significantly more abundant compared to *Tr *were enriched in the non-syntenic areas (Table 9). In addition, the number of paralogous genes was significantly increased in the non-syntenic regions. We considered three possible explanations for this: the non-syntenic genes were present in the last common ancestor of all three *Trichoderma *species but were then selectively and independently lost; the non-syntenic areas arose from the core genome by duplication and divergence during evolution of the genus *Trichoderma*; and the non-syntenic genes were acquired by horizontal transfer. To distinguish among these hypotheses for their origin, we compared the sequence characteristics of the genes in the non-syntenic regions to those present in the syntenic regions in *Trichoderma *and genes in other filamentous fungi. We found that the majority (>78%) of the syntenic as well as non-syntenic encoded proteins have their best BLAST hit to other ascomycete fungi, indicating that the non-syntenic regions are also of fungal origin. Also, a high number of proteins encoded in the non-syntenic regions of *Ta *and *Tv *have paralogs in the syntenic region. Finally, codon usage tables and codon adaptation index analysis [32] indicate that the non-syntenic genes exhibit a similar codon usage (Figure S3 in Additional file 3). Taken together, the most parsimonious explanation for the presence of the paralogous genes in *Ta *and *Tv *is that the non-syntenic genes arose by gene duplication within a *Trichoderma *ancestor, followed by gene loss in the three lineages, which was much stronger in *Tr*.

**Table 9:** Number of PFAM domains that are enriched among paralogous genes in non-syntenic areas

	*T. reesei*	*T. virens*	*T. atroviride*
Zn2Cys6 transcription factors	9	**95**	69
WD40 domains	1	11	14
Sugar transporters	**0**	18	13
Proteases	2	28	23
Cytochrome P450	7	**40**	15
NmrA-domains	2	19	21
Major facilitator superfamily	7	52	60
HET domains	3	26	27
Glycoside hydrolases	3	33	26
FAD-binding proteins	2	28	24
Ankyrins	4	44	37
Alcohol dehydrogenases	**4**	51	71
α/ß-fold hydrolases	2	26	15
ABC transporters	4	14	3
Number of genes	50	485	418
Total gene number in NS areas	92	686	1012

Boxed numbers are those that are significantly (p < 0.05) different from the two other species when related to the genome size. PFAM, protein family; NS, non-syntenic; HET, heteroincompatibility.

**Figure 2:**
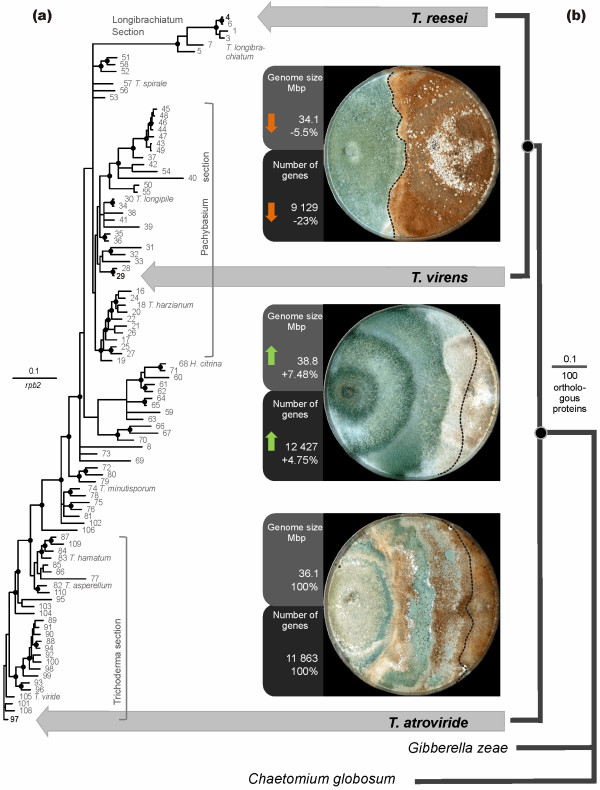
**Mycoparasitism is an ancient life style of *Trichoderma***. **(a) **Position of *Ta, Tv *and *Tr *within the genus *Hypocrea*/*Trichoderma*. The positions of *Tr, Tv *and *Ta *are 4, 29 and 97, respectively - shown in bold), and a few hallmark species are given by their names. For the identities of the other species, see the gene accession numbers (Materials and methods). **(b) **Bayesian phylogram based on the analysis of amino acid sequences of 100 orthologous syntenic proteins (MCMC, 1 million generations, 10,449 characters) in *Tr, Tv, Ta, Gibberella zeae *and *Chaetomium globosum*. Circles above nodes indicate 100% posterior probabilities and significant bootstrap coefficients. The numbers in the boxes between (a) and (b) indicate the genome sizes and gene counts and percentage net gain regarding *Ta*. Photoplates show the mycoparasitic reaction after the contact between *Trichoderma *species and *Rhizoctonia solani*. *Trichoderma *species are always on the left side; dashed lines indicate the position of *Trichoderma *overgrowth of *R. solani*.

*Tr, Ta *and *Tv *each occupy very diverse phylogenetic positions in the genus *Trichoderma*, as shown by a Bayesian *rpb2 *tree of 110 *Trichoderma *taxa (Figure 2). In order to determine which of the three species more likely resembles the ancestral state of *Trichoderma*, we performed a Bayesian phylogenetic analysis [33] using a concatenated set of 100 proteins that were encoded by orthologous genes in syntenic areas in the three *Trichoderma *species and also *G. zeae *and *Chaetomium globosum*. The result (Figure 2) shows that *Ta *occurs in a well-supported basal position to *Tv *and *Tr*. These data indicate that *Ta *resembles the more ancient state of *Trichoderma *and that both *Tv *and *Tr *evolved later. The lineage to *Tr *thus appears to have lost a significant number of genes present in *Ta *and maintained in *Tv*. The long genetic distance of *Tr *further suggests that it was apparently evolving faster then *Ta *and *Tv *since the time of divergence.

To test this assumption, we compared the evolutionary rates of the 100 orthologous and syntenic gene families between the three *Trichoderma *species. The median values of the evolutionary rates (K_s _and K_a_) of *Ta-Tr *and *Tv-Tr *were all significantly higher (1.77 and 1.47, and 1.33 and 1.19, respectively) than those of *Ta-Tv *(1.13 and 0.96; all *P *values <0.05 by the two-tailed Wilcoxon rank sum test). This result supports the above suggestion that *Tr *has been evolving faster than *Ta *and *Tv*.

## Discussion

Comparison of the genomes of two mycoparasitic and one saprotrophic *Trichoderma *species revealed remarkable differences: in contrast to the genomes of other multicellular ascomycetes, such as aspergilli [15,17], those of *Trichoderma *appear to be have the highest level of synteny of all genomes investigated (96% for *Tr *and still 78/79% for *Tv and Ta*, respectively, versus 68 to 75% in aspergilli), and most of the differences between *Ta *and *Tv *versus *Tr *or other ascomycetes occur in the non-syntenic areas. Nevertheless, at a molecular level the three species are as distant from each other as apes from *Pices *(fishes) or *Aves *(birds) [17], suggesting a mechanism maintaining this high genomic synteny. Espagne *et al. *[13] proposed that a discrepancy of genome evolution between *P. anserina, N. crassa *and the aspergilli and saccharomycotina yeasts is based on the difference between heterothallic and homothallic fungi: in heterothallics the presence of interchromosomal translocation could result in chromosome breakage during meiosis and reduced fertility, whereas homothallism allows translocations to be present in both partners and thus have fewer consequences on fertility. Since *Trichoderma *is heterothallic [34], this explanation is also applicable to it. However, another mechanism, meiotic silencing of unpaired DNA [35] - which has also been proposed for *P. anserina *[13], and which eliminates progeny in crosses involving rearranged chromosomes in one of the partners - may not function in *Trichoderma *because one of the essential genes (*SAD2 *[36]) is missing.

Our data also suggest that the ancestral state of *Hypocrea*/*Trichoderma *was mycoparasitic. This supports an earlier speculation [37] that the ancestors of *Trichoderma *were mycoparasites on wood-degrading basidiomycetes and acquired saprotrophic characteristics to follow their prey into their substrate. Indirect evidence for this habitat shift in *Tr *was also presented by Slot and Hibbett [38], who demonstrated that *Tr *- after switching to a specialization on a nitrogen-poor habitat (decaying wood) - has acquired a nitrate reductase gene (which was apparently lost earlier somewhere in the Sordariomycetes lineage) by horizontal gene transfer from basidiomycetes.

Furthermore, the three *Trichoderma *species have the lowest number of transposons reported so far. This is unusual for filamentous fungi, as most species contain approximately 10 to 15% repetitive DNA, primarily composed of TEs. A notable exception is *Fusarium graminearum *[27], which, like the *Trichoderma *species, contains less than 1% repetitive DNA [8]. The paucity of repetitive DNA may be attributed to RIP, which has been suggested to occur in *Tr *[8] and for which we have here provided evidence that it also occurs in *Ta *and *Tv*. It is likely that this process also contributes to prevent the accumulation of repetitive elements.

The gene inventory detected in the three *Trichoderma *species reveals new insights into the physiology of this fungal genus: the strong expansion of genes for solute transport, oxidoreduction, and ankyrins (a family of adaptor proteins that mediate the anchoring of ion channels or transporters in the plasma membrane [39]) could render *Trichoderma *more compatible in its habitat (for example, to successfully compete with the other saprotrophs for limiting substrates). In addition, the expansion of WD40 domains acting as hubs in cellular networks [40] could aid in more versatile metabolism or response to stimuli. These features correlate well with a saprotrophic lifestyle that makes use of plant biomass that has been pre-degraded by earlier colonizers. The expansion of HET proteins (proteins involved in vegetative incompatibility specificity) on the other hand suggests that *Trichoderma *species may frequently encounter related yet genetically distinct individuals. In fact, the presence of several different *Trichoderma *species can be detected in a single soil sample [41]. Unfortunately, vegetative incompatibility has not yet been investigated in any *Trichoderma *species, and based on the current data, should be a topic of future research.

Finally, the abundance of SSCPs in *Trichoderma *may be involved in rhizosphere competence: the genome of the ectomycorrhizal basidiomycete *Laccaria bicolor *also encodes a large set of SSCPs, which accumulate in the hyphae that colonize the host root [42].

Gene expansions in *Tv *and *Ta *that do not occur in *Tr *may comprise genes specific for mycoparasitism. As a prominent example, proteases have expanded in *Ta *and *Tv*, supporting the hypothesis that the degradation of proteins is a major trait of mycoparasites [43]. Likewise, the increase in chitinolytic enzymes and some ß-glucanase-containing GH families is remarkable and illustrates the importance of destruction of the prey's cell wall in this process. With respect to the chitinases, the expansion of those bearing CBM50 modules was particularly remarkable: proteins containing these modules were recently classified into several different groups by de Jonge and Thomma [44]. Proteins that consist solely of CBM50 modules are type-A LysM proteins, and there is evidence for the role of these as virulence factors in plant pathogenic fungi. The high numbers of LysM proteins that are found in *Trichoderma*, however, indicate other/additional roles for these proteins in fungal biology that are not understood yet. Also, the expansion of the GH75 chitosanases was intriguing: chitosan is a partially deacetylated derivative of chitin and, depending on the fungal species and the growth conditions, in mature fungal cell walls chitin is partially deacetylated. It has also been reported that fungi deacetylate chitin as a defense mechanism [45,46]. Chitosan degradation may therefore be a relevant aspect of mycoparasitism and fungal cell wall degradation that has also not been regarded yet. Overall, the carbohydrate-active enzyme machinery present in *Trichoderma *is compatible with saprophytic behavior but, interestingly, the set of enzymes involved in the degradation of 'softer' plant cell wall components, such as pectin, is reduced. A possible plant symbiotic relationship [3] might rely on a mycoparasitic capacity along with a reduced specificity for pectin, minimizing the plant defense reaction.

Although the genes encoding proteins for the synthesis of typical fungal secondary metabolites (PKS, NRPS, PKS-NRPS) are also abundant, they are not significantly more expanded than in some other fungi. An exception is *Tv *and its 28 NRPS genes. However, our genome analysis revealed also a high number of oxidoreductases, cytochrome P450 oxidases, and other enzymes that could be part of as yet unknown pathways for the synthesis of further secondary metabolites. In support of this, several of these genes were found to be clustered in the genome (data not shown), and were more abundant in the two mycoparasitic species *Ta *and *Tv*. Together with the expanded set of oxidoreductases, monooxygenases, and enzymes for AMP activation of acids, phosphopathetheine attachment, and synthesis of isoquinoline alkaloids in *Ta *and *Tv*, these genes may define new secondary metabolite biosynthetic routes.

## Conclusions

Our comparative genome analysis of the three *Trichoderma *species now opens new opportunities for the development of improved and research-driven strategies to select and improve *Trichoderma *species as biocontrol agents. The availability of the genome sequences published in this study, as well as of several pathogenic fungi and their potential host plants (for example, [47]) provides a challenging opportunity to develop a deeper understanding of the underlying processes by which *Trichoderma *interacts with plant pathogens in the presence of living plants within their ecosystem.

## Materials and methods

### Genome sequencing and assembly

The genomes of *T. virens *and *T. atroviride *each were assembled from shotgun reads using the JGI (USA Department of Energy) assembler Jazz (see Table S15 in Additional file 1 for summary of assembly statistics). Each genome was annotated using the JGI Annotation pipeline, which combines several gene prediction, annotation and analysis tools. Genes were predicted using Fgenesh [48], Fgenesh+ [49], and Genewise programs [50]. ESTs from each species (Chapter 4 of Additional file 2) were clustered and either assembled and converted into putative full-length genes directly mapped to genomic sequence or used to extend predicted gene models into full-length genes by adding 5' and/or 3' untranslated regions to the models. From multiple gene models predicted at each locus, a single representative model was chosen based on homology and EST support and used for further analysis. Gene model characteristics and support are summarized in Tables S16 and S17 in Additional file 1.

All predicted gene models were functionally annotated by homology to annotated genes from a NCBI non-redundant set and classified according to Gene Ontology [51], eukaryotic orthologous groups (KOGs) [52], and Kyoto Encyclopedia of Genes and Genomes (KEGG) metabolic pathways [53]. See Tables S18 and S19 in Additional file 1 for a summary of the functional annotation. Automatically predicted genes and functions were further refined by user community-wide manual curation efforts using web-based tools at [54,55]. The latest version gene set containing manually curated genes is called GeneCatalog.

Assembly and annotation data for *Tv *and *Ta *are available through JGI Genome Portals homepage at [54,55]. The genome assemblies, predicted gene models, and annotations were deposited at GenBank under project accessions [GenBank: ABDF00000000 and ABDG00000000], respectively. GenBank public release of the data described in this paper should coincide with the manuscript publication date.

### Genome similarity analysis and genomic synteny

Orthologous genes, as originally defined, imply a reflection of the history of species. In recent years, many studies have examined the concordance between orthologous gene trees and species trees in bacteria. With the purpose of identifying all the orthologous gene pairs for the three *Trichoderma *species, a best bidirectional blast hit approach as described elsewhere [56,57] was performed, using the predicted translated gene models for each of the three species as pairwise comparison sets. The areas of relationship known as syntenic regions or syntenic blocks are anchored with orthologs (calculated as mutual best hits or bi-directional best hits) between the two genomes in question, and are built by controlling for the minimum number of genes, minimum density, and maximum gap (genes not from the same genome area) compared with randomized data as described in [56]. While this technique may cause artificial breaks, it highlights regions that are dynamic and picking up a large number of insertions or duplications.

Orthologous and paralogous gene models were identified by first using BLAST to find all pairwise matches between the resulting proteins from the gene models. The pairwise matches from BLAST were then clustered into groups of paralogs using MCL [58]. In parallel we applied orthoMCL [59] to the same pairwise matches to identify the proteins that were orthologous in all of the three genomes. By subtracting all the proteins that were identified as orthologs from the groups of paralogs and unique genes, we were left with only the protein products of gene models that have expanded since the most recent common ancestor (MRCA) of the three *Trichoderma *genomes. We then calculated the *P*-value under the null hypothesis that the number of non-orthologous genes that are non-syntenic is less than the number of non-orthologous genes that are syntenic.

### Identification of transposable elements

We scanned the *Trichoderma *genomes with the *de novo *repeat finding program Piler [19]. Next, we searched for sequences with similarity to known repetitive elements from other eukaryotes with the program RepeatMasker [21] using all eukaryotic repetitive elements in the RepBase (version 13.09) database. After masking repetitive sequences that matched the DNA sequence of known repetitive elements, we scanned the masked genome sequences with RepeatProteinMask (a component of the RepeatMasker application). This search located additional degenerate repetitive sequences with similarity to proteins encoded by TEs in the RepBase database.

### CAZome identification and analysis

All protein models for *Ta *and *Tv *were compared against the set of libraries of modules derived from CAZy [60,61]. The identified proteins were subjected to manual analysis for correction of the protein models, for full modular annotation and for functional inference against a library of experimentally characterized enzymes. Comparative analysis was made by the enumeration of all modules identified in the three *Trichoderma *species and 14 other published fungal genomes.

### Phylogenetic and evolutionary analyses

One-hundred genes were randomly selected from *Ta, Tv, Tr *and *C. globosum *based on their property to fulfill two requirements: they were in synteny in all four genomes, and they were true orthologues (no other gene encoding a protein with amino acid similarity >50% present). After alignment, the concatenated 10,449 amino acids were subjected to Bayesian analysis [33] using 1 million generations. The respective cDNA sequences (31,347 nucleotides) were also concatenated, and Ks/Ka ratios determined using DNASp5 [62]. The same file was also used to determine the codon adaptation index [32]. In addition, 80 non-syntenic genes were also selected randomly for this purpose.

The species phylogram of *Trichoderma*/*Hypocrea *was constructed by Bayesian analysis of partial exon nucleotide sequences (824 total characters from which 332 were parsimony-informative) of the *rpb2 *gene (encoding RNA polymerase B II) from 110 *ex*-type strains, thereby spanning the biodiversity of the whole genus. The tree was obtained after 5 million MCMC generations sampled for every 100 trees, using burnin = 1200 and applying the general time reversible model of nucleotide substitution. The NCBI ENTREZ accession numbers are: 1 [HQ260620]; 3 [DQ08724]; 4 [HM182969]; 5 [HM182984]; 6 [HM182965]; 7 [AF545565]; 8 [AF545517]; 16 [FJ442769]; 17 [AY391900]; 18 [FJ179608]; 19 [FJ442715]; 20 [FJ442771]; 21 [AY391945]; 22 [EU498358]; 23 [DQ834463]; 24 [FJ442725]; 25 [AF545508]; 26 [AY391919]; 27 [AF545557]; 28 [AF545542]; 29 [FJ442738]; 30 [AF545550]; 31 [AY391909]; 32 [AF545516]; 33 [AF545518]; 34 [AF545512]; 35 [AF545510]; 36 [AF545514]; 37 [AY391921]; 38 [AF545513]; 39 [AY391954]; 40 [AY391944]; 41 [AF545534]; 42 [AY391899]; 43 [AY391907]; 44 [AF545511]; 45 [AY391929]; 46 [AF545540]; 47 [AY391958]; 48 [AY391924]; 49 [AF545515]; 50 [AY391957]; 51 [AF545551]; 52 [AF545522]; 53 [FJ442714]; 54 [AF545509]; 55 [AY391959]; 56 [DQ087239]; 57 [AF545553]; 58 [AF545545]; 59 [DQ835518]; 60 [DQ835521]; 61 [DQ835462]; 62 [DQ835465]; 63 [DQ835522]; 64 [AF545560]; 65 [DQ835517]; 66 [DQ345348]; 67 [AF545520]; 68 [DQ835455]; 69 [AF545562]; 70 [AF545563]; 71 [DQ835453]; 72 [FJ179617]; 73 [DQ859031]; 74 [EU341809]; 75 [FJ179614]; 76 [DQ087238]; 77 [AF545564]; 78 [FJ179601]; 79 [FJ179606]; 80 [FJ179612]; 81 [FJ179616]; 82 [EU264004]; 83 [FJ150783]; 84 [FJ150767]; 85 [FJ150786]; 86 [EU883559]; 87 [FJ150785]; 88 [EU248602]; 89 [EU241505]; 90 [FJ442762]; 91 [FJ442741]; 92 [FJ442783]; 93 [EU341805]; 94 [FJ442723]; 95 [FJ442772]; 96 [EU2415023]; 97 [EU341801]; 98 [EU248600]; 99 [EU341808]; 100 [EU3418033]; 101 [EU2485942]; 102 [AF545519]; 103 [EU248603]; 104 [EU248607]; 105 [EU341806]; 106 [DQ086150]; 107 [DQ834460]; 108 [EU711362]; 109 [EU883557]; 110 [FJ150790].

## Abbreviations

CAZy: Carbohydrate-Active enZYmes; CBM: carbohydrate binding module; EST: expressed sequence tag; GH: glycosyl hydrolase; HET: heteroincompatibility; KEGG: Kyoto Encyclopedia of Genes and Genomes; KOG: clusters of eukaryotic orthologous groups; NRPS: non-ribosomal peptide synthase; PKS: polyketide synthase; RIP: repeat-induced point mutation; SSCP: small secreted cysteine-rich protein; *Ta*: *Trichoderma atroviride*; TE: transposable element; Tr: *Trichoderma reesei*; Tv: *Trichoderma virens*.

## Authors' contributions

CPK, IVG, BH, EM, SEB, CMK, and AHE contributed equally to this work as senior authors. AA, JC, MM, AS, and IVG performed global annotation and analysis, MZ and HS did the assembly, OC and CH finished the assembly, and EL and SL performed the genome and EST sequencing. SEB, AH-E, CMK and CPK designed the study, and coordinated and supervised the analysis; CPK drafted and submitted the paper. All other authors contributed research (annotations and/or analyses). All authors read and approved the final manuscript.

## Supplementary Material

Additional file 1 **Comparative properties and gene inventory of *T. reesei, T. virens *and *T. atroviride***. This file contains additional information on genomic properties and selected gene families from the three *Trichoderma *species comprising 19 tables. Table S1 summarizes the satellite sequences identified in the *Trichoderma *genomes and four other fungal genomes. Table S2 summarizes manually curated sequence alignments of transposable element families from the *Trichoderma *genomes. Table S3 lists the total number of CAZy families in *Trichoderma *and other fungi. Table S4 lists the glycoside hydrolase (GH) families in *Trichoderma *and other fungi. Table S5 lists the glycosyltransferase (GT) families in *Trichoderma *and other fungi. Table S6 lists the polysaccharide lyase (PL) families in *Trichoderma *and other fungi. Table S7 lists the carbohydrate esterase (CE) families in *Trichoderma *and other fungi. Table S8 lists the carbohydrate-binding module (CBM) families in *Trichoderma *and other fungi. Table S9 lists the NRPS, PKS and NRPS-PKS proteins in *T. atroviride*. Table S10 lists NRPS, PKS and NRPS-PKS proteins in *T. virens*. Table S11 lists the putative insecticidal toxins in *Trichoderma*. Table S12 lists the cytochrome P450 CYP4/CYP19/CYP26 class E proteins in *Trichoderma*. Table S13 lists the small-cysteine rich secreted protein from *Trichoderma *spp. Table S14 lists the most abundant PFAM domains in those genes that are unique to *T. atroviride *and *T. virens *and not present in *T. reesei*. Table S15 surveys the assembly statistics. Table S16 provides gene model support. Table S17 summarizes gene model statistics. Table S18 provides numbers of genes with functional annotation according to KOG, Gene Ontology, and KEGG classifications. Table S19 lists the largest KOG families responsible for metabolism. Click here for file

Additional file 2 **Additional information on selected gene groups of *Trichoderma*, methods used for genome sequencing, and legends for the figures in Additional file **3. Chapter 1: Carbohydrate-Active enzymes (CAZymes). Chapter 2: Aegerolysins and other toxins. Chapter 3: Small secreted cysteine rich proteins (SSCPs). Chapter 4: EST sequencing and analysis. Chapter 5: Legends to figures. Click here for file

Additional file 3 **Figures that illustrate selected aspects of the main text**. Figure S1 provides a phylogeny of *Trichoderma *NPRSs. Figure S2 compares the numbers of epoxide hydrolase genes in *Trichoderma *with that in other fungi. Figure S3 compares the codon usage in genes from syntenic and nonsyntenic regions of the genomes of *Trichoderma reesei, T. atroviride *and *T. virens*. Click here for file
