# Supplementary material for: Comparative genome sequence analysis underscores mycoparasitism as the ancestral life style of Trichoderma
Source: Genome Biol. 2011 Apr 18;12(4):R40. doi: 10.1186/gb-2011-12-4-r40 (PMC3218866; doi:10.1186/gb-2011-12-4-r40)
Supplement: Additional file 2 — Additional information on selected gene groups of Trichoderma, methods used for genome sequencing, and legends for the figures in Additional file 3. Chapter 1: Carbohydrate-Active enzymes (CAZymes). Chapter 2: Aegerolysins and other toxins. Chapter 3: Small secreted cysteine rich proteins (SSCPs). Chapter 4: EST sequencing and analysis. Chapter 5: Legends to figures. [file gb-2011-12-4-r40-S2.DOCX]

**Comparative genome sequence analysis underscores mycoparasitism as the ancestral life style of *Trichoderma***

**Additional File 2**

1. Carbohydrate Active enzymes (CAZymes)

2. Aegerolysins and other toxins

3. Small secreted cysteine rich proteins (SSCPs)

4. EST sequencing and analysis

5. Legends to Supplementary Figures

**1. Carbohydrate Active enzymes (CAZymes)**

Carbohydrate-active enzymes or CAZymes are categorized into different families in the CAZy database [60,61]. These families describe structurally-related catalytic and carbohydrate-binding modules of enzymes that degrade, modify, or create glycosidic bonds. The categories in the CAZy database include glycoside hydrolases (GHs), glycosyltransferases (GTs), polysaccharide lyases (PLs), carbohydrate esterases (CEs) and carbohydrate binding modules (CBMs). We examined the CAZyme genome content (CAZome) of *T. virens* and *T. atroviride* and compared it with the corresponding gene subsets from 15 other fungi (11 ascomycetes and 3 basidiomycetes) whose genomes have been sequenced.

In comparison to *T. reesei*, which is surprisingly poor in genes encoding glycoside hydrolases (GHs), a total number of 259 and 258 GH-encoding genes for *T. virens* and *T. atroviride* respectively, ranks them on the forefront for ascomycetes. With 98 and 100 glycosyltransferases (GTs) in *T. atroviride* and *T. virens*, they are close to *T. reesei* (103) and slightly above the average of the analysed fungi (95). The similar numbers of GTs that are found in different fungi are a probable consequence of the conserved role of the vast majority of GTs in housekeeping and intracellular communication activities. The number of carbohydrate esterases (CEs) and polysaccharide lyases (PLs) in *Trichoderma* was generally lower compared to other fungi in our dataset, but clearly higher in *T. atroviride* and *T. virens* than in *T. reesei*. Further, in contrast to *T. reesei*, whose genome has the smallest number of CBM-containing proteins among the Sordariomycetes, *T. virens* had the second highest number of CBMs. The number of CBMs in *T. atroviride* was within the average of Sordariomycetes.

*T. reesei* is an efficient degrader of cellulosic plant matter, but its pool of cellulases (covering part of the GH5 proteins identified, and all of GH6, 7, 12, 45, 61) and xylanases and associated hemicellulase activities (GH10, 11, 26, 29, 39, 62, 67, 74, 93) is relatively small in comparison to other ascomycetes. The number of identified cellulases and xylanases has not significantly expanded in *T. virens* and *T. atroviride*, suggesting that the observed low variety of cellulases and xylanases is a common feature of the genus *Trichoderma*. Furthermore, a reduced capacity to interact with pectin is observed, with a relatively low presence of catalytic modules in families GH28, 78, 88, and 105, families PL1, 3 and 4, and families CE8 and 12, all directly acting on polygalacturonan and rhamnogalacturonan or in the respective degradation products. Equally, the number and nature of pectin side-chain degrading enzymes, involving proteins from families GH43, 51, 53, and 54, is equally reduced in all *Trichoderma*, when compared to plant cell-degrading *Ascomycetes*. A reduced capacity to degrade “soft” plant cell wall components, although still compatible with a saprophytic life-style, could be particularly relevant for a symbiotic life style with plants for *Trichoderma* species.

In addition to chitin, glucans, especially β-1,3- and α-1,3/1,4-glucans, and galactomannans are major components of fungal cell walls [64]. Accordingly, further GH families that are expanded in *Trichoderma* spp. are, GH55 and GH64, (all containing β-1,3-glucanases), GH27 (α-galactosidases) and GH92 (α-mannosidases). β-1,3- and α-glucan form the amorphous cement of the fungal cell wall of *Aspergilli* and the major alkali-soluble polysaccharide of the cell wall [65]. Genes encoding α-1,3-glucan synthases, which are bifunctional enzymes containing an intracellular GT5 and an extracellular GH13 module, are omnipresent in a large taxonomical range of fungi but completely absent from all three sequenced *Trichoderma* strains. The apparent absence of α-1,3/1,4-glucan synthases in *Trichoderma* points to a different cell wall composition in this genus. Interestingly, *Trichoderma* spp. possess several members of GH71, which are α-1,3-glucanases. The absence of GT5/GH13 α-glucan synthases found for *Trichoderma* and *Fusarium* appears to correlate with the presence of abundant GT64 candidate α-glycosyltransferases, whose function is uncharacterized in fungi, but that are absent for all organisms containing α-glucan synthases. A possible α-glucan degrading capacity could be complemented by the existing activity of amylolytic enzymes from families GH13 and 15.

Further GH families that are enriched in *Trichoderma* spp. are GH54 (known for α-arabinofuranosidase activity), GH65 (α-diglucoside phosphorylases, trehalases), GH79 (β-glucuronidases), GH95 (α-1,2-L-fucosidases). Gene expression of several members of the above mentioned families was upregulated in *T. atroviride* and partially in *T. virens* during mycoparasitism, indeed indicating a link between an expansion of these families and their relevance for mycoparasitism. This demonstrates that besides the so far known enzymatic players of the mycoparasitic attack, namely chitinases and β-1,3-glucanases, there are a number of other enzymatic activities involved in this process which were not known so far to be of importance for degradation of the fungal cell envelope.

A notable difference between *T. reesei* and the mycoparasites *T. atroviride* and *T. virens* is that the latter two possess an invertase (GH32). This is in agreement with the finding that *T. reesei* cannot grow on sucrose as carbon source [66], but is also important in defining crucial aspects of *Trichoderma*-plant interactions. It was recently shown in *T. virens* that plant-derived sucrose is associated with the control of root colonization and that the fungal intracellular invertase is crucial for the mechanisms that control the symbiotic association and fungal growth in the presence of sucrose [67]. The saccharolytic activity in the fungal cells affects the sink activity of roots, directing carbon partitioning toward roots and increased the rate of photosynthesis in leaves of maize (*Zea mays*).

The pectin degradation abilities of *Trichoderma* spp. appear very poor in comparison to other fungi; e.g. *A. niger* has already at least 39 genes encoding enzymes involved in the depolymerisation of the backbone of pectin [68]. Additional genes encoding enzymatic activities required for the degradation of the arabinan and arabinogalactan sidechains were also detected. In contrast to that, *T. reesei* has only a total 6 pectinolytic enzymes, whereas *T. virens* and *T. atroviride* have a somewhat better pectin degradation machinery and possess at least 15 pectinolytic enzymes. A number of pectin degrading enzymes (GH78, 88, 105, PL1, CE8) are enriched in these two fungi in comparison to *T. reesei*. These additional proteins could constitute a small albeit important difference facilitating the attachment to roots or the displacement of hyphae in the pectin-rich intercellular space of plants, thereby deepening fungal-plant interactions of *T. virens* and *T. atroviride.*

CBMs are often found as modules of enzymes involved in plant polysaccharide depolymerization appended to the catalytic domain. They aid in attachment of the enzymes to their respective substrates and prevent dissociation of the enzymes and the carbohydrate polymer after the hydrolytic step, thus enabling a processive degradation of the substrate. However, there are also several proteins in the *Trichoderma* genomes that consist solely of CBMs. Although the function of these proteins remains to be elucidated, it is possible that these proteins act as accessory proteins, which are potential facilitators of carbohydrate polymer degradation because they increase the substrate accessibility of enzymes by loosening up the rigid structure of the biomass fibers, it is equally feasible that their repeated nature helps in masking cell-wall chitin and preventing the action of the chitinases present in the environment. In total, 17 % of *T. virens* GHs and 14 % of *T. atroviride* GHs possess CBMs and interestingly many of them were found in GH families that were enriched in the mycoparasites, especially GH 18 (chitinases), but also GH54 (α-arabinofuranosidases) and GH65 (α-diglucoside phosphorylases and trehalases) or in families that were found to be upregulated during the mycoparasitic attack, GH71 (α-1,3-glucanases) and CE4 (chitin deacetlyases).

Finally, all three *Trichoderma* spp. also are enriched in families PL 7 and 8. The enzymatic activities of these families that described so far are alginate lyase (PL7) and chondroitin/hyaluronan lyase (PL8). Only few other fungi possess proteins of these families, whereas *T. atroviride* and *T. virens* have each 3 members of PL7 and 1 of PL8. The precise activity of fungal PL7/ 8 enzymes is not known yet. Phylogenetic analysis of these proteins showed that they lacked close neighbors in other fungi, and bear high similarity to bacterial proteins, and it is thus possible that their genes were obtained by horizontal gene transfer. Their potential roles in mycoparasitism remain to be elucidated. However, PL7/8 enzymes could also be involved in the interaction with bacteria and respective biofilms or could also be important for the growth and defense of living space of *Trichoderma* in the soil since these fungi are not only mycoparasites but are also able to survive in the soil. Significantly, the presence of non-plant cell-wall aiming polysaccharide lyases has equally been noticed in the plant mycorrhizal symbiont *Laccaria bicolor* [42].

**2. Aegerolysins and other toxins**

The aegerolysins are a class of genes expressed during formation of primordia and fruiting bodies, and that they may play a role in the initial phase of fruiting in basidiomycetes [69]. The bacterial members of this family, on the other hand, are expressed during sporulation. Ostreolysin was found cytolytic to various erythrocytes and tumour cells, because they form transmembrane pores 4 nm in diameter.

Genes encoding putative aegerolysins were only found in *T. virens* (Trive1:67227) and *T. atroviride* (Triat1:32864), but not in *T. reesei*. They exhibited low similarity to *A. fumigatus* and *A. oryzae*  (8e-24 and 6e-23, respectively), but were absent from all other ascomycetes for which genome sequences are available.

We also found two set of genes encoding high molecular weight toxins in *T. atroviride* and *T. virens*. The putative proteins bear high similarity to the Tc-(toxin complex) toxins - approximately 1 MDa protein complexes which are toxic to insect pests - of *Photorhabdus luminescens*, a bacterium which is mutualistic with entomophagous nematodes, and which secretes this toxin into the insect hemocoel upon nematode invasion [70]. Subsequent genome sequencing projects have revealed the presence of Tc orthologues in a range of bacterial pathogens known to be associated with insects. In other bacteria such as *Yersinia pseudotuberculosis,* these toxins were shown to have evolved to confer mammalian pathogenicity [71].

In addition to *Trichoderma*, we found a copy of the toxin 1 genes in *F. graminearum, Nectria haematococca, Podospora anserina and Verticillium alboatrum*, suggesting its presence in the Sordariomycetes. BLAST analysis and Phylogenetic analysis implies proteins from Myxobacteria and Actinomycetes as the next neighbour, but the branch leading to these two clades is only poorly supported. Interestingly, the fungal proteins are still flanked by sequences with high similarity to the *Salmonella* SpvB [72] and *Escherichia coli* RhsA [73] proteins suggesting the possibility of their origin in fungi by ancient horizontal gene transfer. *T. virens* contains 4 paralogs of this gene.

The toxin 2 genes showed a similar limited distribution among the Sordariomycetes, but proteins with high similarity were found also in *Botryotinia fuckeliana* (coverage 75 %, E value 0) and *Sclerotinia sclerotiorum* (coverage 70 %, E value 0). The C-terminal half of the proteins exhibited high similarity to the insectidical toxin of *Photorhabdus* RHA1 and unknown proteins from *Sorgangium cellulosum* and *Plesiocystis pacifica* (5.6e^-128^ – 4e^-105^). The presence of these two potential insecticidal toxin genes in mycoparasitic *Trichoderma* spp. renders it also possible that the two species may be active against insects.

**3. Small secreted (cysteine-rich) proteins (SSCRPs)**

For a first survey of the SSPs of *T. virens, T. atroviride*, and *T. reesei*, a set of best filtered protein models from JGI was the initiation point. From these, predicted proteins less than or equal to 300 amino acids long and containing four or more cysteine residues were selected. The size cutoff criterion was chosen as described [74], and the presence of four cysteines screened based on our candidate proteins and those in the literature. The resulting lists were screened for signal peptides in batch mode at the SignalP server [75], selecting those predicted to have a signal peptide according to HMM. Entries with clear homology to known enzymes, as well as proteins annotated by the JGI pipeline as containing transmembrane segments, were excluded by manual inspection of the lists. Several hundred candidate SSPs were identified by this procedure in each species. It is important to note that this survey provides a subset of the predicted secretome, which will need to be analyzed more completely in subsequent studies. The expansin-like protein swollenin [76], for example, is larger than the 300 aa cutoff value. To classify the SSCPs, they were analyzed for similarity using BLASTclust [77] at coverage 80% and an identity cutoff of 20%. This analysis resulted in 24 clusters containing 4 or more members, numbered from largest to smallest; some of these served to generate phylogenies across the three species using PHYML (data not shown). A large fraction of the SSCPs in each species, however, had no homologs at the cutoff level chosen, in the same or in the other two species. This result implies that there is a striking level of diversity between the three species, suggesting that SSCPs are evolving rapidly. SSCPs might, therefore, provide good candidates for biotechnological applications for improved systemic resistance and biocontrol of pathogens.

We used TBLASTN [74] to search each candidate SSCP in the translated nucleotide databases of the other two species. When those with homologs of BLAST scores of >200 were excluded, the numbers of predicted unique SSCPs are reduced to 83 in *T. atroviride*, 75 in *T. virens* and 38 in *T. reesei*. The two mycoparasites, *T. atroviride* and *T. virens*, have about two-fold more unique SSCPs than *T. reesei*, perhaps reflecting the interaction of *T. atroviride* and *T. virens* with a wide range of fungal host and plant species. 68 of the SSCPs are shared, at 20% identity, between all three *Trichoderma* species.

Cluster 1, the largest, contains 32 predicted proteins of unknown function. Cluster 2 includes the Class II hydrophobins. Hydrophobins are SSCPs characterized by an 8-cysteine signature. They coat the fungal cell surface at the interface between air and a water-saturated substrate. Hydrophobins thus have roles in development and infection [78, 79]. Comparison with the annotation of Class II in the three species [80] shows that the majority of the Class II hydrophobin genes that we detected are known hydrophobins. In addition, the SSCP screen identified another hydrophobin group in *T. atroviride* and *T. virens*, but not in *T. reesei*. This group provides new candidates for Class I hydrophobins in *Trichoderma*. Ta55150, belonging to Cluster 21, shows homology to a *T. asperellum* hydrophobin involved in plant root colonization [81], which is the only Class I hydrophobin detected in *Trichoderma* hitherto. In a few cases, some known Class II hydrophobin genes were not identified by the SSCP screen. Conversely, three Class II hydrophobins are predicted by the SSCP screen but were not previously annotated. Each such case will need to be addressed individually in the three species.

Cluster 4 includes Sm1, required for induction of ISR [82-84] and also a second branch defined by Snodprot homologs (i.e ceratoplatanins). The predicted product of the MAPK-regulated gene *mrsp1* [85] belongs to a small group, Cluster 12. Consideration of the relationships defined by phylogeny of the SSPs will help design experiments to elucidate the functions of the novel clusters, and of novel members of clusters that include previously studied genes.

#### 4. EST sequencing and Analysis.

#### *T. virens* nuclei were isolated using a modification of a previously published protocol [86]. Mycelia were harvested by filtration (10 g wet weight) and washed with 10 mM Tris-HCl buffer (pH 7.5). The washed mycelia were frozen in liquid nitrogen and ground to a fine powder with a mortar and pestle. The powder was resuspended in 8 ml of Buffer A (1 M Sorbitol, 7% Ficoll w/v, 20% glycerol, 50 mM Tris-HCl (pH7.5), 0.5% Triton X-100) by stirring for 5 min on ice. The crude homogenate was filtered through miracloth (Calbiochem) and 2 volumes of Buffer B (25 mM Tris-HCl, (pH7.5), 10% glycerol, 0.5% Triton X-100) was added with stirring on ice. Five ml of a 5:8 mixture of Buffer A: Buffer B was placed in a 50 ml centrifuge tube and the homogenate was carefully layered on top, followed by centrifugation at 3000 x g for 7 min at 4° in the SW28 rotor to remove debris. The supernatent was layered onto 5 ml of Buffer C (25 mM Tris-HCl (pH 7.5), 10% glycerol, 1 M sucrose) in a fresh tube and centrifuged at 9400 x g for 15 min to pellet the nuclei. The supernatent was removed and the nuclear pellet was resuspended in 10 ml of TE (10 mM Tris-HCl (pH 7.5), 1 mM EDTA) and transferred to a 50 ml polypropylene tube. Ten ml of phenol: chloroform was added and the mixture was rocked for 5 min to allow mixing. After a brief centrifugation, the supernatent was transferred to a fresh tube and phenol: chloroform extraction was repeated until precipitation was no longer observed. The supernatent was then transferred to a fresh tube, overlayed with 2 volumes of 95% ethanol, and the DNA was spooled onto a glass rod. The spooled DNA was dissolved in TE with DNAse-Free RNAse (10 ug/ml) for 1 h at 37° and then precipitated by addition of sodium acetate to 0.3 M and 2 volumes of 95% ethanol. The DNA pellet was resuspended to 1 mg/ml for further analysis.

#### For preparation of ESTs for *T. virens*, RNA was isolated from fungal tissue grown under eight different conditions in liquid culture shaking at 125 rpm at 27^o^ C in an incubator. For all eight conditions, conidia (1x 10^6^/ml) of Gv29-8 were inoculated into Vogel’s minimal medium (VM, [87]) supplemented with 1.5% sucrose (VMS) and incubated for 48 hrs. Tissue was harvested, rinsed in sterile distilled water, and equal weight of tissue added to each of eight different conditions. These conditions included: VM + *Rhizoctonia solani* cell wall material, incubated 2 hrs; VM + *R. solani* cell wall material, incubated 24 hrs; VM + *Pythium ultimum* cell wall material, incubated 24 hrs; Weindling’s medium supplemented with arginine; incubated for 24 hrs; VM with no C or N source, incubated 2 hrs; VM with no C or N source, incubated 24 hrs; VM + sterile cotton roots (dried and ground), incubated 2 hrs; and VM + sterile cotton roots (dried and ground), incubated 24 hrs. RNA was extracted by method of Jones et al. [88].

#### *T*. atroviride was grown on Vogel's minimal medium with 0.3% (w/v) glucose as carbon source. For confrontation assays agar plates were overlaid with cellophane and inoculated with an agar plaques of the plant pathogen (R. solani, B. cinerea), and T. atroviride in approximately 5 cm distance from the pathogen. Strains were grown in complete darkness at 28°C and T. atroviride was harvested under red safety light at pre-contact (1-2 mm distance of the mycelia) and immediately frozen in liquid nitrogen. For light induced conidiation, T. atroviride cultures were grown in the dark for 48 h at 27°C on PDA plates and used as pre-inoculum. Mycelial plugs (0.5 cm diam) were taken from the colony growth front and placed on the centre of plates containing Vogel's medium covered with a cellophane membrane. Cultures were allowed to grow for further 36 h under these conditions, and then photoinduced as described [89] by exposure to white light for 5 min (fluence rate 27 mmol m^-2^ s^-1^). For injury-induced conidiation, fungal colonies were grown in total darkness on PDA at 27°C for 72 h, cut in stripes with a scalpel and incubated for an additional 24 h in the dark at 27°C. For mere mycelial growth, T. atroviride was grown on PDA plates in complete darkness at 28°C for 48 hrs.

#### *For* cDNA library sequencing, Bacterial colonies containing either *T. virens* or T. atroviride cDNA library were plated onto agarose plates (254 mm plates from Teknova, Hollister, CA) at a density of approximately 1000 colonies per plate. Plates were grown at 37 C for 18 hours then individual colonies were picked and each used to inoculate a well containing LB media with appropriate antibiotic in a 384 well plate (Nunc, Rochester, NY). Clones were grown in 384 well plates at 37 C for 18 hours. Contained plasmid DNA for sequencing was produced by rolling circle amplification [90] (Templiphi, GE Healthcare, Piscataway, NJ). Inserts were sequenced from both ends using primers complimentary to the flanking vector sequence, and Big Dye terminator chemistry then run on ABI 3730 instruments (ABI, Foster City, CA). The sequencing primers used were the following: *T. atroviride*: for the mycelial injury library (vector: pDONR222; Fw: 5'-GTAAAACGACGGCCAGT, Rv: 5'-AGGAAACAGCTATGACCAT), for the light exposed library and the mycoparasitism library (vector: pEXP-AD502; Fw: 5' CTATTCGATGATGAAGATACC Rv: 5' AGAAGTCCAAAGCTCCACC), and for the dark exposed library (vector: pSPORT1; 5'-GTTTTCCCAGTCACGACGTTGTA, Rv: 5'-AGGAAACAGCTATGACCAT). *T. virens*: for the libraries (vector:  pCMVSPORT6 (Invitrogen); Fw: 5’ ATTTAGGTGACACTATAGAA Rv: 5’ TAATACGACTCACTATAGGG).

#### ESTs were processed through the JGI EST pipeline (ESTs were generated in pairs, a 5' and 3' end read from each cDNA clone). To trim vector and adaptor sequences, common sequence patterns at the ends of ESTs (Expressed Sequence Tags) were identified and removed using an internally developed software tool. Insertless clones were identified if either of the following criteria were met: >200 bases of vector sequence at the 5' end or less than 100 bases of non-vector sequence remained. ESTs were then trimmed for quality using a sliding window trimmer (window = 11 bases). Once the average quality score in the window was below the threshold (Q15) the EST was split and the longest remaining sequence segment was retained as the trimmed EST. EST sequences with less than 100 bases of high quality sequence were removed. ESTs were evaluated for the presence of polyA or polyT tails (which if present were removed) and the EST re-evaluated for length, removing ESTs with less than 100 bases remaining. ESTs consisting of more than 50% low complexity sequence were also removed from the final set of "good ESTs". In the case of re-sequencing the same EST, the longest high quality EST was retained. Sister ESTs (end pair reads) were categorized as follows: if an EST lacked an insert or was a contaminant, then by default the second sister was categorized as the same. However, each sister EST was treated separately for complexity and quality scores. Finally, EST sequences were compared to the GenBank/EMBL/DDBJ nucleotide database in order to identify contaminants; non-desirable ESTs such as those matching non-cellular and rRNA sequences were removed.

For clustering, ESTs were evaluated with MALIGN, a k-mer based alignment tool, which clusters ESTs based on sequence overlap (k-mer = 16, seed length requirement = 32, alignment ID ≥ 98%). Clusters of ESTs were further merged based on sister ESTs using double linkage. Double linkage requires that 2 or more matching sister ESTs exist in both clusters to be merged. EST clusters were then each assembled using CAP3 [91] to form consensus sequences. Clusters may have more than one consensus sequence for various reasons to include; the clone has a long insert, clones are splice variants or consensus sequences are erroneously not assembled. Cluster singlets are clusters of one EST, whereas CAP3 singlets are single ESTs which had joined a cluster, but during cluster assembly were isolated into a separate singlet consensus sequence. ESTs from each separate cDNA library were clustered and assembled separately and subsequently the entire set of ESTs for all cDNA libraries were clustered and assembled together.

**5. Legends to Supplementary Figures**

**Supplementary Figure S1**

Genealogy of *Trichoderma* NPRSs, inferred by maximum parsimony analysis of the A domain (AMP binding adenylate domain). Major clades and subclades are indicated by vertical bars, each of which shares a common organization of domains. Numbers above branches indicate percentage bootstrap support for each clade (numbers below 50% are not shown). The sequences of the A domain (AMP binding adenylate domain) were aligned with CLUSTALW and manually adjusted, subsequently alignment was analysed also by COBALT multiple sequence alignment tool that finds a collection of pairwise constraints derived from conserved domain database, protein motif database, and sequence similarity, using RPS-BLAST, BLASTP, and PHI-BLAST. Pairwise constraints are then incorporated into a progressive multiple alignment [92]. The final resulted alignment exported in fasta format was phylogenetically analyzed. The genealogy of 213 A domains obtained from 84 NRPS and 7 Hybrid PKS-NRPS was inferred by maximum parsimony analysis using the PAUP Version 4.0b10 (Sinauer Associates, Sunderland, Massachusetts). Alignment was based on 653 aminoacids from the A domain; 466 characters were parsimony-informative characters; 72 characters were constant and 115 characters are parsimony-uninformative. Bootstrap method with heuristic search and number of bootstrap replicates = 500. All positions containing gaps and missing data were eliminated from the dataset (Complete Deletion option).

**Supplementary Figure S2**

Number of epoxide hydrolase gene models in *Trichoderma* and other fungi

**Supplementary Figure S3**

Comparison of codon usage in genes from syntenic and nonsyntenic regions of the genomes of *T. reesei, T. atroviride* and *T. virens*. (a) Plot of usage in nonsyntenic (NS) vs. syntenic (S) genes. Values of usage of one of the codons are given between 0 and 1, the latter specifying that only a single codon is used. R is the Pearson correlation coefficient for correlation between the data sets. (b) Codon adaptation index for the NS and S genes for the three species.
