# Supplementary material for: Comparative genome sequence analysis underscores mycoparasitism as the ancestral life style of Trichoderma
Source: Genome Biol. 2011 Apr 18;12(4):R40. doi: 10.1186/gb-2011-12-4-r40 (PMC3218866; doi:10.1186/gb-2011-12-4-r40)
Supplement: Additional file 3 — Figures that illustrate selected aspects of the main text. Figure S1 provides a phylogeny of Trichoderma NPRSs. Figure S2 compares the numbers of epoxide hydrolase genes in Trichoderma with that in other fungi. Figure S3 compares the codon usage in genes from syntenic and nonsyntenic regions of the genomes of Trichoderma reesei, T. atroviride and T. virens. [file gb-2011-12-4-r40-S3.DOC]

**Comparative genome sequence analysis underscores mycoparasitism as the ancestral life style of *Trichoderma***

**Additional File 3**

Figure S1**.**

Figure S2

**Number of epoxide hydrolases**

Figure S3.
